# Q Fever Outbreak in Homeless Shelter

**DOI:** 10.3201/eid1007.031020

**Published:** 2004-07

**Authors:** Philippe Brouqui, Sékéné Badiaga, Didier Raoult

**Affiliations:** * Université de la Méditérranée, Marseille, France;; † Centre Hospitalier Universitaire Nord Marseille, Marseille, France

**Keywords:** homeless, Q fever, Coxiella burnetii, slaughterhouse, wind, dispatch

## Abstract

Urban outbreaks of Q fever have occurred after exposure to slaughterhouses or parturient cats. We detected an outbreak of Q fever in a homeless shelter in Marseilles. Investigations showed that the main factors exposing persons to *Coxiella burnetii* were an abandoned slaughterhouse, used for an annual Muslim sheep feast, and wind.

Homelessness, a problem that has been increasing since the mid-1980s, raises substantial public health concerns (1). We have worked with the homeless population of Marseilles since 1993 in ongoing studies on louse-transmitted diseases (2,3).

Q fever is a zoonosis caused by *Coxiella burnetii*, an intracellular bacterium transmitted by aerosols from contaminated soil or animal waste or by drinking contaminated milk (4). Domestic ungulates (cows, sheep, and goats) are the main reservoir for *C. burnetii*, but other mammals, including dogs, cats, and wild rabbits, have been implicated (5). Q fever usually occurs in rural areas when people are incidentally exposed to aerosols or infected milk or milk products (6), although urban outbreaks have been reported after occupational exposure to slaughterhouses (7–9).

While investigating louse-borne diseases in two homeless shelters in Marseilles, we systematically tested persons for antibodies to *C. burnetii* and found a significantly higher seroprevalence in the homeless population from the northern shelter (Figure 1) than in control blood donors. This shelter (A) is located 2 kilometers south of an abandoned slaughterhouse that is used 1 day each year by the Muslim population of Marseilles for the traditional sheep feast, "Aid El Khebir," during which sheep are ritually killed. We hypothesized that the northern wind (the mistral) that blows over the slaughterhouse and the shelter was involved in spreading *C. burnetii*, as has been reported previously in another outbreak near Marseilles (10). We consequently investigated and followed-up the homeless population from the two shelters for 4 consecutive years and report here the first outbreak of Q fever in this population. We propose that the wind played a critical role in the outbreak.

**Figure 1:**
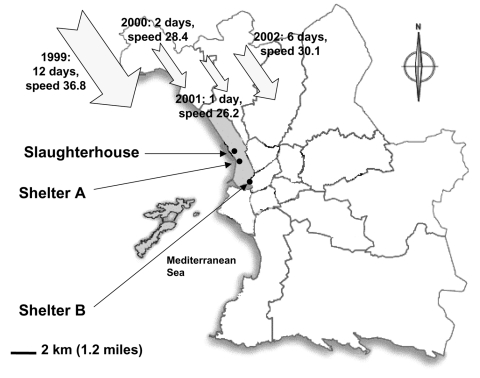
Study sites in Marseilles. The cumulative number of days, strength of the mistral measured as a mean of the daily recorded maximum mistral speed in km/h, and direction of the wind during the month that followed the Aid El Khebir, shown by year.

## The Study

The protocol was reviewed and approved by an institutional review board (Comités Consultatifs de Protection des Personnes dans la Recherche Biomédicale 99/76), and all participants gave informed consent. A medical team of 27 persons, comprising 9 nurses, 6 infectious diseases residents or fellows, and 12 infectious diseases specialists, visited the two shelters once yearly for 4 consecutive years. Each shelter can accommodate 300 persons each night, and each offers showers, food, washing machines, and clean clothes. Shelter B is located downtown, while shelter A is located in the northern part of the town (Figure 1). Homeless persons completed a standardized questionnaire, and a physical examination was performed. Nurses collected blood samples for laboratory investigation. Control subjects were sex- and age-matched blood donors enrolled during the same period and living in Marseilles. Serologic analysis was carried out at the French National Reference Center for Rickettsial Diseases. The antigen used was a phase II and phase I *C. burnetii* Nine Mile strain (ATCC VR 615) grown in our laboratory in L929 mouse fibroblasts. Phase I was obtained by injection in mice. Samples were assessed by microimmunofluorescence (MIF) as described elsewhere (11). Immunoglobulin (Ig) G phase II antibody titer >1:50 indicated *C. burnetii* exposure in the past 6 months to 5 years.

Meteorologic data were obtained from Meteo-France departmental weather stations (http://www.meteo.fr/meteonet/meteo/pcv/cdm/dept13/cdm2.htm#3). Maximum wind speeds and directions were measured three times each hour, which led to >1,400 data entries for the month observed. We asked for wind information during the month which followed the Aid El Khebir in each year: March 27–April 27, 1999; March 16–April 16, 2000; March 6–April 6, 2001; and February 23–March 23, 2002. Epidemiologic, clinical, and laboratory data were entered into SPSS Data Entry Builder 3.0 (SPSS Inc., Chicago, IL) and then analyzed with SPSS 10.0 (SPSS Inc.). Wind data were captured in an Excel database (Microsoft, Redmond, WA). Qualitative variables were compared with Fisher or χ^2^ tests. A logistic regression model was used for multivariate analysis. The regression was carried out stepwise, and the model included all variables present in the univariate analysis for which p < 0.20.

A total of 930 homeless persons were recruited, 261 in 2000, 171 in 2001, 296 in 2002, and 202 in 2003. Mean age was 43 years (range 18–83), with 44 females and 886 males. Country of origin was France for 36.7%, northern Africa for 37%, eastern Europe for 15.4%, western Europe for 6.2%, sub-Saharan Africa for 2.4%, and Asia for 0.7%. Among 467 controls, 217 completed the questionnaire.

*C. burnetii* IgG phase II antibodies were found in 17 (10.8%) of 157 persons in shelter A in 2000, compared to 14 (3%) of 460 controls (p < 0.001). In this shelter, the number of patients with a positive test result was significantly higher in 2000 than in 2001 (0/96, 0%) and 2002 (1/182, 0.54%) (p < 0.001). This difference was not significant when compared with results in 2003 (7/129, 5.4%). The number of homeless persons with positive test results for *C. burnetii* was not significantly different between shelter B residents and controls (Table).

**Table:** Homeless persons positive for *Coxiella burnetii* phase II antibodies > 1:50 compared to controls^a^

Group	Positive serologic test results for Q fever, positive/tested (%)			
	2000	2001	2002	2003
Shelter A residents	17/157 (10.8)^b^	0/96 (0)^b^	1/182 (0.54)^b^	7/129 (5.4)
Shelter B residents	2/104 (1.9)	0/75 (0)	0/114 (0)	0/73(0)
Controls	14/460 (3)^b^	NA	NA	NA

^a^NA, not applicable. ^b^p < 0.001.

When exposure to cats, kittens, or dogs; Muslim religion; and living in shelter A were considered, only contact with a kitten (p = 0.031) was associated with *C. burnetii* positivity in the univariate analysis. However, multivariate analysis using a stepwise linear regression model with all variables included in the univariate analysis showed that living in shelter A was the only factor independently associated with a positive test result for *C. burnetii*. Moreover, one person in 2002 and seven in 2003 were found positive in shelter A, compared to none in shelter B in those years. Acute Q fever was diagnosed in three homeless persons with IgM anti–phase II antibodies >1:50, one person in 2000 and two in 2003. Two were asymptomatic, and one showed symptoms of high-grade fever, arthralgia, myalgia, and dyspnea. He was hospitalized, and a chest x-ray noted interstitial bilateral pneumonitis. No cardiac murmur was detected. The serologic tests showed IgG, IgM, and IgA antibody titers of 1:800, 1:50, and 1:200 to phase II antigen and 1:400, 1:25, and 1:200 to phase I antigen, respectively. He was treated with 200 mg oral doxycycline each day for 15 days, and he recovered.

Weather records showed that the cumulative number of windy days with the wind blowing from the north (N), north-northwest (NNW), and northwest (NW) was significantly higher in the month that followed the Aid El Khebir in 1999 compared to 2000 (12/32, p = 0.002) and 2001 (2/32, p = 0.0006) (Figure 1) but not to 2002. The strength of the mistral measured as a mean of the daily recorded maximum speed was not significantly different among the investigated years (Figure 1).

## Conclusions

Q fever is a disease caused by *C. burnetii*, a strict intracellular bacterium that can survive in the environment for up to 10 months at 15°-20°C, for >1 month on meat in cold storage, and for >40 months in skim milk at room temperature (5). Two distinct sets of symptoms of Q fever are prevalent. In the acute phase, patients may have fever, granulomatous hepatitis, or interstitial pneumonitis; the chronic phase is primarily characterized by culture-negative endocarditis. The acute phase is asymptomatic in >50% of cases, which explains why an outbreak might be unnoticed (9,12).

**Figure 2:**
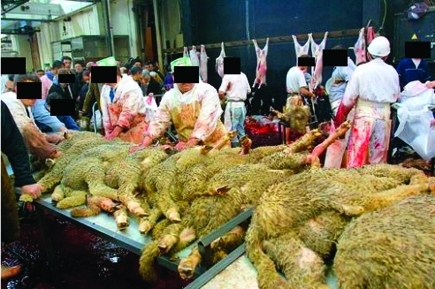
The Aid El Khebir sheep sacrifice in the abandoned Marseilles slaughterhouse.

Q fever is primarily transmitted to humans when aerosolized fluids are inhaled during or after parturition of an infected animal. The organism can stick on wool and dust and be spread by wind. The wind has been shown to spread *C. burnetii* in other circumstances. In a small town in southern France, wind blew through a steppe where sheep were gathered after lambing, and persons whose homes were exposed to the wind were more often infected with Q fever than their neighbors (10). In cities, the role of slaughterhouses in the spread of Q fever is well-known (7,8). The last reported slaughterhouse-related outbreak of Q fever in France was related to contaminated waste from sheep sacrificed for a Christian Easter feast. The waste had been left uncovered outside the slaughterhouse, which was near a heliport. Helicopters might have facilitated airborne transmission of the infectious agent (9). *C. burnetii* has also been shown to be transmitted by dogs (13), wild rabbits (14), and parturient cats (15), and transmission has been associated with religious practices (16). In this study, contact with kittens and correlation with wind from the slaughterhouse were the only identified risk factors. We showed here that homeless persons were likely exposed to *C. burnetii* in shelter A during the month that followed the Aid El Khebir in 1999, with the wind playing a critical role in this outbreak. Some controversy surrounds this feast in France because of the way sheep are ritually sacrificed. Several hundred sheep are maintained for a few days inside and outside the slaughterhouse before having their throats slit and being bled outside. They are then displayed to buyers, as shown in Figure 2. That sheep are maintained under conditions of poor hygiene, without veterinary counsel, and that the bleeding and sale takes place outside may explain how *C. burnetii*–infected particles could have contaminated soil, wool, or loose straw, and particles could have blown downwind. Veterinary control of sheep flocks would help avoid such contamination.

Shelter B is further south than shelter A. Since no significant differences in incidence of Q fever were found in shelter B, homelessness itself is not associated with Q fever. Access to health care is problematic for this population, so an outbreak of Q fever could go unnoticed unless Q fever testing was a part of disease surveillance in homeless persons. The risk of an unnoticed outbreak emphasizes the need to systematically survey this population and nearby residents. Persons in other areas surrounding the slaughterhouse were also likely exposed to *C. burnetii* in 1999.
